# Large-scale turbulence structures in a laboratory-scale boundary layer under steady and gusty wind inflows

**DOI:** 10.1038/s41598-019-45873-x

**Published:** 2019-06-28

**Authors:** W. J. Li, Y. Zhang, B. Yang, J. W. Su, Y. W. Zhang, W. Z. Lu, Q. X. Shui, X. Y. Wu, Y. P. He, Z. L. Gu

**Affiliations:** 1 School of Human Settlements and Civil Engineering, Xi’an Jiaotong University, Xi’an, 710054 P.R. China; 2 School of Energy and Power Engineering, Xi’an Jiaotong University, Xi’an, 710049 P.R. China; 3 School of Chemical Engineering, Northwest University, Xi’an, 710127 P.R. China; 4 Department of Architecture and Civil Engineering, City University of Hong Kong, Kowloon Tong, 999077 Hong Kong

**Keywords:** Fluid dynamics, Atmospheric dynamics

## Abstract

Experiments on turbulence structures and features of a wind field under steady inflow and gusty wind inflows were implemented in a straight-through wind tunnel. Streamwise and wall-normal velocity components were measured using a streamline constant temperature anemometer (streamline CTA). Power spectra analyses revealed the existence of very large-scale motions (VLSMs) under both steady and gusty wind inflows; but new gusty scale motions (GSMs) were revealed under only gusty wind inflows. The GSMs might originate from an ordered external driving force that forces hairpin packets to align coherently in groups with a length scale related to the gust inflow condition. The streamwise wavelength of VLSMs is independent of inflow conditions, while the turbulent energy of VLSMs is associated with the wall-normal height and local mean streamwise velocity. In particular, the streamwise wavelength of GSMs increases linearly with the average value and period of sinusoidal gusty wind inflows, and the turbulent energy of GSMs is sensitive to the wall-normal height and all characteristic parameters of gusty wind inflows, including the average value, amplitude and period. Considerable wall-normal airflows induced by gusty wind inflows were detected and these are negatively correlated with the variation in gusty streamwise velocity, and root mean square (RMS) values of the gusty wall-normal velocity tended to increase linearly with the average value and amplitude of gusty wind inflows.

## Introduction

Over the past few decades, considerable attention has focused on understanding the turbulence structures of wall-bounded turbulent flows, which undeniably play a universal and important role in many engineering and scientific applications^1–4^. Wall-bounded turbulence is composed of certain recurrent features termed ‘coherent structures’. These structures have various length scales and therefore carry different portions of the turbulent kinetic energy (TKE). Recently, large-scale coherent structures termed large-scale motions (LSMs) with lengths of 2*δ*~3*δ* in the streamwise direction were observed in turbulent boundary layer flows^5,6^, channel flows^7^ and pipe flows^8^. Here, *δ* is the boundary layer thickness, the channel half-height, or the pipe radius *R*. Much longer highly elongated structures of uniform streamwise momentum regarded as very large-scale motions (VLSMs) or superstructures (SS) were also detected in turbulent boundary layer flows^9,10^ and pipe flows^8,11,12^. These results indicate that LSMs and VLSMs are energetic and typically contain half of the TKE of the streamwise component^13^.

Most research on turbulence structures has been based on steady inflows^5–12^. Even field investigations have been carried out by selecting measured data with fine stability to avoid the complexity and uncontrollability of environmental conditions^6,10^. In fact, vast observation data of air flows show that rather regular low-frequency fluctuations with a period of 1–10 min, termed gusty wind disturbances^14,15^, are superimposed on the basic flow in some weather conditions, such as the passage of a cold front. The characteristics of these fluctuations are very different from those of classic turbulent fluctuations with relatively high frequency^16–19^. According to World Meteorological Organization (WMO) definition^15^, the gust decay time (*t*_*d*_) and the time internal between gust and lull (*t*_*gl*_) are some gusty characteristics. Usually, *t*_*d*_ > 1 min, and *t*_*gl*_ is about several minutes. This means that gusts as disturbances are in the period domain [1 min, 10 min].

The horizontal velocity spectra acquired by Xu *et al*.^16^ and Cheng *et al*.^14^ demonstrated the scales and TKE of turbulent eddies during periods of gusty wind were much larger than those before the outbreak of gusty wind. This was a qualitative study on the turbulence structures under gusty wind conditions; but further quantitatively is necessary to investigate the scale and TKE of the turbulence structures under gusty wind inflows, and the influence of various characteristic parameters of gusty wind on the scale and TKE distribution of the turbulence structures. In addition, numerous studies have demonstrated that the gustiness of the wind velocity could play a key role in the transport of matter and energy in the turbulent boundary layer^20–22^. The simulation results of Cheng *et al*.^20^ indicated that the coherent structure of the gusty wind can effectively entrain sand particles into the middle and upper levels of the atmospheric boundary layer. A numerical simulation of the wind field in a street canyon under gusty wind inflow^21^ illustrated an obvious intermittent feature and flapping of the shear layer, resulting in expansion or compression of the main vortex in the street canyon. Duan and Ngan^22^ incorporated time-periodic perturbations in the streamwise velocity component, and a small but statistically significant response to inflow perturbations was subsequently observed in the turbulent flow inside an ideal street canyon. Hence, it is of great significance to study the coherent structures and wind fields of the turbulent boundary layer under the inflow superimposed by gusty wind disturbances.

Scientific research usually employ at least one of three methods: field observation, numerical simulation and experimental research. Because of the uncontrollability of natural flow conditions, field observations can only be conducted qualitatively to reveal the basic form and features of turbulence structures and cannot quantitatively analyse the influence of various characteristic parameters of gusty disturbances. With respect to numerical simulation, direct numerical simulation (DNS) can calculate all scales of turbulences but is restricted to situations with very low Reynolds numbers. Experimental research in a wind tunnel could be a useful approach to qualitatively and quantitatively study wind fields and turbulence structures of the turbulent boundary layer under gusty wind inflows. In this study, measurements of air flows in a straight-through wind tunnel were implemented under steady inflow and gusty wind inflows. The gusty wind inflows were produced by automatically adjusting the frequencies of inlet and outlet blowers in a sine law, and the streamwise and wall-normal velocity components of the air flow in the wind tunnel were measured by a streamline constant temperature anemometer (streamline CTA) equipped with a two-dimensional probe. Then, spectral analyses of measured data were carried out to explore the turbulence structures and TKE distribution. Due to the limitation of experimental facilities, only sinusoidal gusty wind inflow can be realized in our experiments, which seems not to match the actual situation. While, any set of wind speed data can be decomposed into multiple sets of sinusoidal wind speed data by fast Fourier transformation (FFT). Consequently, the sinusoidal gusty wind inflow can be adopted as the most basic object of the study to reveal turbulence structures and wind field features under gusty wind inflows. Furthermore, the sinusoidal gusty wind inflow is more convenient to quantitatively study the influence of various characteristic parameters of gusty disturbances than natural gusty wind inflow.

This paper is arranged as follows. Sec. 2 describes the experimental set-up. Tests of the mean streamwise velocity profile and features of the turbulent boundary layer under different running frequencies of the blowers are presented in Sec. 3. The experimental results are analysed in Sec. 4. Sec. 5 gives the conclusions.

## Experimental Set-up

The experiments were conducted in a straight-through wind tunnel with a working section of 20 × 0.6 × 0.8 m at Northwest University, Shaanxi Province, China. One blower with a maximum running frequency of 50 Hz is located at both the entrance and exit of the wind tunnel. In contrast to the blower running mode typically used in wind tunnels, the blowers in our wind tunnel were equipped with a frequency conversion control platform to allow the blowers to run either in fixed frequency mode or in sinusoidal frequency mode with a minimum period of 20 s, which can be expressed as

1 $${f}_{b}=\{\begin{array}{ll}{\rm{const}}, & {\rm{fixed}}\,{\rm{frequency}}\,{\rm{mode}}\\ \overline{{f}_{b}}+{A}_{b}\,\sin (\frac{2\pi }{{T}_{b}}t) & {\rm{sinusoidal}}\,{\rm{frequency}}\,{\rm{mode}}\end{array}$$

where *f*_*b*_ is the running frequency of the blowers, which is a constant for the fixed frequency mode, but varies sinusoidally with time for the sinusoidal frequency mode, $\overline{{f}_{b}}$, *A*_*b*_ and *T*_*b*_ are respectively the average value, amplitude and period of the sinusoidal running frequency. On account of the proportional relation between the rotational speed and running frequency of the blowers, *r* = 60 * *f*_*b*_/*p*, where *r* is the rotational speed, *p* is the number of the pole pair. The streamwise velocity at a certain point in the wind tunnel varies sinusoidally when the blowers run in sinusoidal frequency mode. Gusty wind inflows are thus produced by automatically adjusting the running frequencies of both blowers synchronously (${f}_{b}^{etrance}={f}_{b}^{exit}$, where ${f}_{b}^{etrance}\,\,$and ${f}_{b}^{exit}$ are the running frequency of the blower at the entrance and exit, respectively.) in sinusoidal frequency mode.

**Figure 1:**
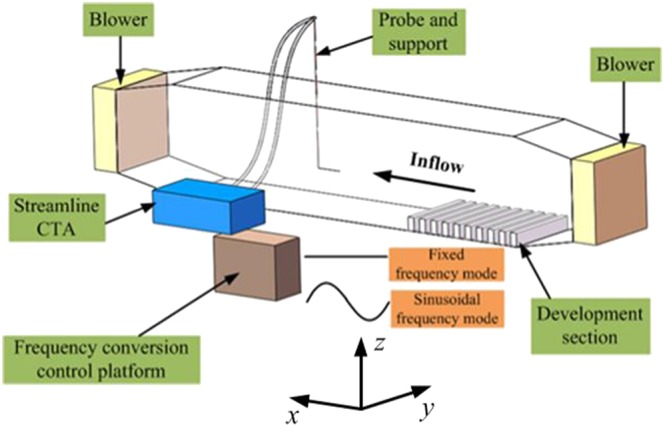
The sketch of wind tunnel experimental system.

Measurements were performed at a distance of 10 m downstream from the entrance, where the turbulent boundary layer is fully developed^23,24^. The development section was constituted by wooden cubical blocks with dimensions 0.6 m (length) × 0.015 m (width) × 0.02 m (height), which were aligned perpendicular to the flow with an interval of 0.1 m. One Dantec 55P61 X-array dual-sensor wire probe was connected with a streamline CTA to obtain streamwise and wall-normal velocity components (normal to wind tunnel floor) at a sample frequency of 100 Hz. The 55P61 probe has 1.25 mm-long platinum-plated tungsten wire sensors with a diameter of 5 μm and 8.4 mm-long straight prongs. The wind speed measurement range of the 55P61 probe is from 0 to 200 m · s^−1^, with a minimum resolution of 0.01 m · s^−1^. The angle between the flow vector and streamwise direction need to be within [−45°, 45°]. The probe was placed on the central plane in the width direction with the leading edge holding horizontally. A sketch of the wind tunnel experiment system is shown in Fig. 1.

## Validation of the Mean Streamwise Velocity Profile

**Figure 2:**
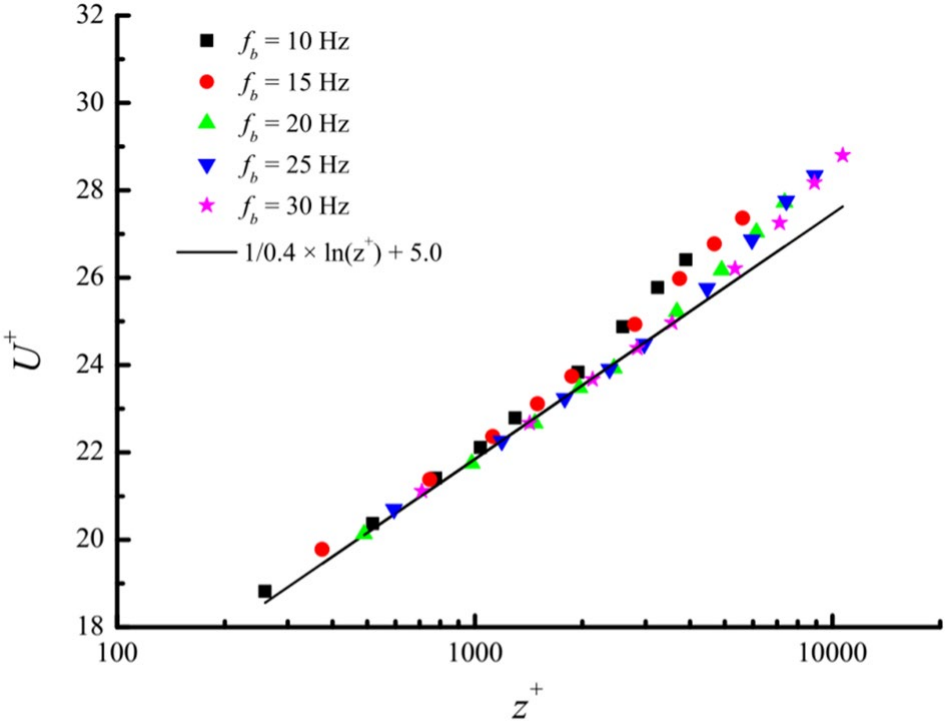
The mean streamwise profiles.

**Figure 3:**
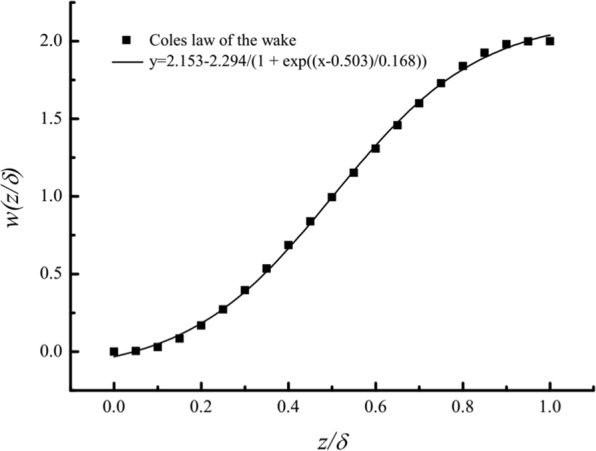
Fitting of the Coles law of the wake.

To determine the basic experimental parameters of our laboratory-scale turbulent boundary layer, the profiles of the mean streamwise velocity in the wind tunnel under steady inflows were first calibrated. Measurements were carried out with both blowers running jointly with the same fixed frequency, *f*_*b*_, of 10, 15, 20, 25 or 30 Hz. The measured non-dimensional mean streamwise velocities, *U*^+^ = *U*/*u*_*τ*_, at different heights are displayed in Fig. 2, where *U* is the mean streamwise velocity with a sampling time interval of 60 s, *u*_*τ*_ is the friction velocity calculated from a Clauser chart fit^25^ with the von Kármán constant *κ* = 0.4 and *A* = 5.0. At the lower position, all profiles agree with the logarithmic law. This region is termed the logarithmic region, and the mean streamwise velocity profile above this region can be described by

2 $${U}^{+}=\frac{1}{\kappa }\,\mathrm{ln}({z}^{+})+B+\frac{{\rm{\Pi }}}{\kappa }w(z/\delta )$$

where *z*^+^ = *zu*_*τ*_/*ν* with kinematic viscosity ν = 1.55 × 10^−5^ m^2^/s is determined by fluid temperature, *B* is a constant to be determined experimentally, Π is a profile parameter that is nearly 0.55 for flow at constant pressure, and *w*(*z*/*δ*) is the wake function given by Coles^26^ in the form of discrete points as shown in Fig. 3. The Coles law of the wake can be fit well with a Boltzmann function. In this study, the boundary layer thickness *δ* is obtained from the fitting wake function^26–28^.

The experimental parameters under steady inflows are listed in Table 1, where *Re*_*τ*_ = *u*_*τ*_*δ*/*ν* is the friction Reynolds number and *U*_*ht*15_ is the mean streamwise velocity at a height of 0.15 m. Because the influence of gust characteristic parameters on turbulence structures and the wind field features take the results at a height of 0.15 m as an example in a later section, *U*_*ht*15_ was adopted as the reference for inflow wind speed. Table 1 shows that *U*_*ht*15_ is a linear function of the running frequency of the blowers, *f*_*b*_, which can be described as

3 $${U}_{ht15}=0.29+0.49{f}_{b}$$

**Table 1:** Experimental parameters under steady inflows.

*f*_*b*_/Hz	*U*_*ht*15_/m · s^−1^	*u*_*τ*_/m · s^−1^	*δ*/m	*Re*_*τ*_
10	5.09	0.20	0.28	3605
15	7.88	0.29	0.26	4883
20	9.88	0.38	0.30	7306
25	12.35	0.46	0.26	7763
30	15.05	0.55	0.28	9829

## Results and Analysis

In nature, gusty wind disturbances are superimposed on the basic flow under certain weather conditions. The characteristics of these disturbances are very different from classic turbulent fluctuations^14^. As mentioned above, the sinusoidal gusty wind inflow can be adopted as the most basic object of our study to reveal the turbulence structures and wind field features under gusty wind inflows. The sinusoidal gusty wind inflows in our wind tunnel were realised by automatically adjusting the frequencies of both blowers synchronously in the sinusoidal frequency mode. As a contrast, the two blowers were run jointly with the same fixed frequencies to produce steady inflows. In the experiments, the streamwise and wall-normal velocity components were measured under steady inflows and gusty wind inflows, respectively.

This section presents the comparison of the power spectrum and wind field features under different inflow conditions to explore the effect of gusty wind disturbances. Furthermore, the influence of various characteristic parameters of the gusty wind disturbances on the turbulence structure and root mean square (RMS) of the wall-normal velocity component are discussed. The characteristic parameters of the gusty wind disturbances include average value, amplitude and period.

### Contrast between steady inflow and gusty wind inflow

**Figure 4:**
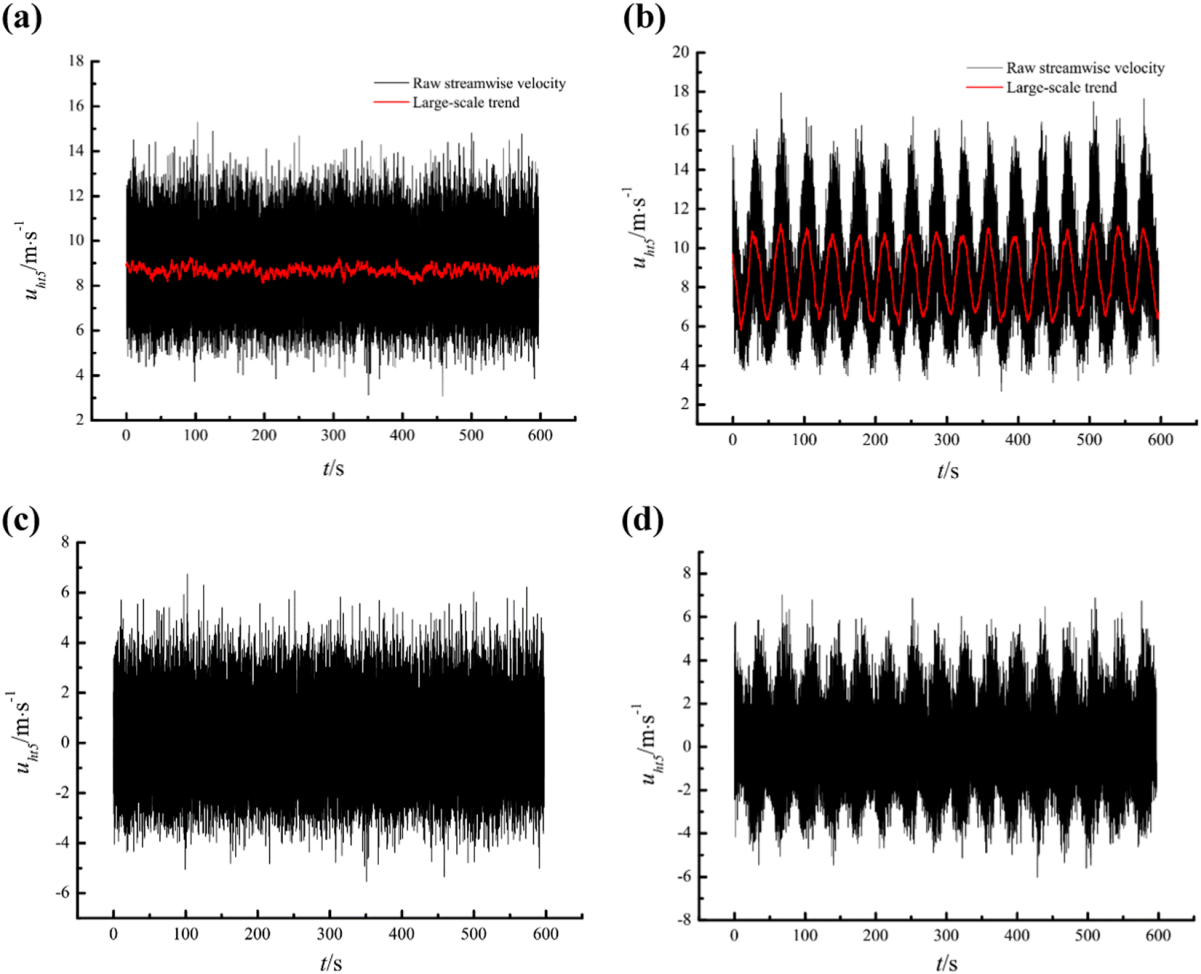
De-trending process: **(a)** the black line is the raw streamwise velocity for steady inflow, and the red line is the large-scale trend for steady inflow; **(b)** the black line is the raw streamwise velocity for gusty wind inflow, and the red line is the large-scale trend for gusty wind inflow; **(c)** de-trended streamwise velocity for steady inflow; **(d)** de-trended streamwise velocity for gusty wind inflow. *u*_*ht*5_ is the streamwise velocity at *z*/*δ* = 0.17.

Gusty wind inflow was realised by automatically adjusting the frequencies of both blowers synchronously in a sine law with an average value $\overline{{f}_{b}}$ = 20 Hz, amplitude *A*_*b*_ = 5 Hz, and period *T*_*b*_ = 60 s, which is greater than the minimum period of the blowers and fall in the range of gusty wind disturbances. As a contrast, measurements under steady inflow with a constant running frequency of the blowers at *f*_*b*_ = 20 Hz were also implemented. The raw streamwise velocity data at 5 heights (*z*/*δ* = 0.17, 0.33, 0.50, 0.67, and 0.83) were obtained under the above mentioned inflow conditions. As an example, the raw streamwise velocity data at *z*/*δ* = 0.17 are shown in Fig. 4(a,b). In addition, Hutchins *et al*.^6^ declared that estimates of turbulence structures can be extremely sensitive to long-term trends (non-turbulence related) in the data. In our experiments, such trends are caused by the sinusoidally changing frequency dominated by frequency conversion control platform and the self-oscillating instability of blowers. The former is the main factor for the data measured under gusty wind inflows. Before the power spectra analyses, a de-trending process is required to eliminate the interference of the non-turbulence-related trends. The results of Hutchins and Marusic^5^ suggest that VLSMs of length 10*δ*–20*δ* are common in laboratory-scale turbulent boundary layers. To retain the VLSMs, the cut-off wavelength can not be less than 20*δ*. Here, the non-turbulence-related trends were acquired through a low-pass filter with a cut-off wavelength of 20*δ*. Then, the streamwise velocity data were de-trended by subtracting this large-scale trend from the raw streamwise velocity data. The de-trended streamwise velocity data at *z*/*δ* = 0.17 are shown in Fig. 4(c,d).

**Figure 5:**
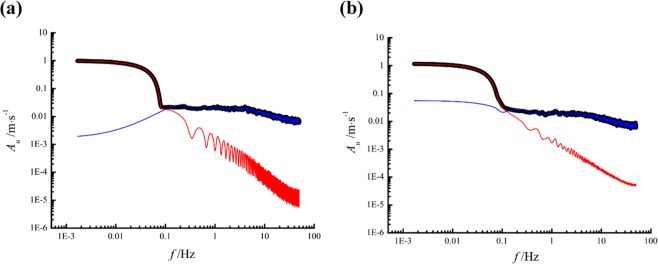
Frequency spectra: **(a)** steady inflow; **(b)** gusty wind inflow. The bold black line represents the result of the raw streamwise velocity data, the red line is the large-scale trend, and the blue line is the de-trended streamwise velocity data. *A*_*u*_ is the velocity amplitude at a certain frequency.

To determine whether the non-turbulence related large-scale trend filtered out or not, frequency spectrum analysis was examined. Figure 5 shows that the frequency spectrum at the lower frequency band for the large-scale trend is completely consistent with that for the raw streamwise velocity data. This comparison proves that the large-scale trend is removed from the raw streamwise velocity data. All subsequent power spectra analyses are based on the de-trended streamwise velocity data.

**Figure 6:**
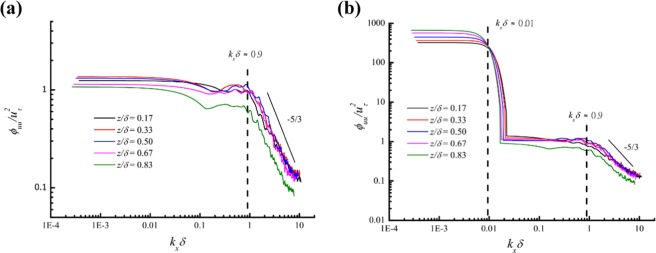
Power spectra of streamwise velocity fluctuations: **(a)** steady inflow; **(b)** gusty wind inflow.

On the basis of the de-trended streamwise velocity, the power spectral density function *ϕ*_*uu*_ was estimated by Welch’s^29^ average periodogram method with a Hanning window and overlapped segment (10% of the window length). Figure 6 shows the normalized spectra ${\varphi }_{uu}/{u}_{\tau }^{2}$ for the steady inflow and gusty wind inflow at 5 heights. Taylor’s hypothesis of frozen turbulence *k*_*x*_ = 2*πf*/*U*_*c*_ was adopted to transform the spectral argument from the radian frequency *ω* = 2*πf* to the streamwise wavenumber *k*_*x*_, where *f* is the sampling frequency of 100 Hz and *U*_*c*_ is the local convection velocity, which is assumed to be equal to the local mean streamwise velocity^30^, *U*. For the steady inflow, all spectral curves have an apparent inflection point (*k*_*x*_*δ* ≈ 0.9) at which the slope of the spectral curve changes obviously. In other words, there are turbulence structures of uniform streamwise momentum in the region of *k*_*x*_*δ* < 0.9 at all 5 heights. According to the streamwise wavelength *λ*_*x*_ = 2*π*/*k*_*x*_, the minimum length scale of these turbulence structures is approximately 7*δ*, and these structures are regarded as VLSMs^9,10^, which is an inherent feature of the turbulent boundary layer flow, independent on the processing method of the raw data. While the de-trended process is necessary to avoid wrongly taking the non-turbulence-related trends as VLSMs. The experiments conducted by Hutchins & Marusic^9^ in an open return suction-type boundary-layer wind tunnel of working section 4.7 × 1.2 × 0.3 m^3^ indicated the streamwise length of VLSMs was around 6*δ* similar to our results. Furthermore, the streamwise length scale of VLSMs is independent on the vertical distance between the measuring point and the floor, which is also consistent with findings acquired by Wang *et al*.^10^ on a dry flat bed of Qingtu Lake in Minqin (China).

For the gusty wind inflow, there is another inflection point (*k*_*x*_*δ* ≈ 0.01) in addition to the one observed in the spectral curves for the steady inflow. The minimum length scale of these larger turbulence structures is approximately 600*δ*, which is close to the order of magnitude of the gusty wind inflow wavelength (~2000*δ*). Here, we call the turbulence structures in the region of *k*_*x*_*δ* < 0.01 as gusty scale motions (GSMs).

Because the de-trending process is implemented through the low-pass filter with a cut-off wavelength of 20*δ*, the structures with the length scale of ~600*δ* are regarded as turbulence structure, which is an inherent feature of the turbulent boundary layer flow independent of the processing method of the raw data. Kim and Adrian^30^ conjectured that hairpins spontaneously align coherently in groups to form long packets, which align coherently to form VLSMs in a spontaneous process. Under gusty wind inflow, the downstream flow field is equivalently applied with an ordered external driving force that actuates very large-scale turbulence structures and/or hairpin packets to align coherently in groups with a length scale related to the inflow condition. This might be a plausible explanation of the formation of GSMs.

**Figure 7:**
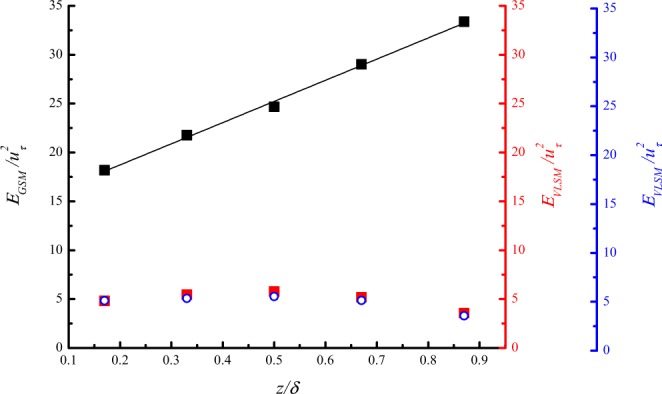
The turbulent energy at 5 heights: the black squares represent the turbulent energy of GSMs for gusty wind inflow; the red squares represent the turbulent energy of VLSMs for gusty wind inflow; the blue circles represent the turbulent energy of VLSMs for steady inflow.

For gusty wind inflow, the turbulent energy *E*_*GSM*_ of the GSMs at 5 heights was calculated through a low-pass filter with a cut-off length of 600*δ*, and a band-pass filter with a cut-off length of 7*δ*~600*δ* was adopted to calculate the turbulent energy of VLSMs, *E*_*VLSM*_. The turbulent energy *E*_*VLSM*_ of VLSMs for steady inflow was also estimated using low-pass filtering with a cut-off length of 7*δ*. Figure 7 demonstrates that the turbulent energy of GSMs is about 3–6 times larger than that of VLSMs, and *E*_*GSM*_ increases linearly with the wall-normal distance. Based on the conjecture that GSMs are formed by the driving of ordered external force, and VLSMs are formed spontaneously, it is reasonable that the turbulent energy of GSMs is much greater than that of VLSMs. The gusty wind inflow would transport energy to the GSMs. In addition, the turbulent energy of VLSMs for both inflow conditions is consistent but is affected to some extent by the wall-normal distance between the measuring points and the ground.

**Figure 8:**
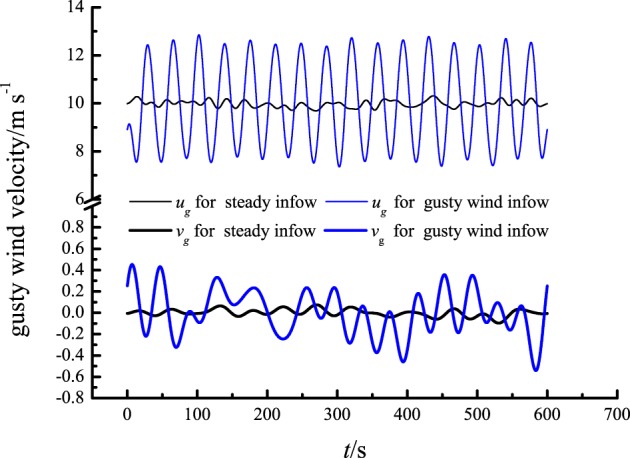
The gusty streamwise and wall-normal velocity components at *z*/*δ* = 0.50, where *u*_*g*_ is the gusty streamwise velocity and *v*_*g*_ is the gusty wall-normal velocity.

Furthermore, Zeng *et al*.^31^ revealed that gusty horizontal wind superimposed on basic wind flow would induce macroscopic vertical airflow with a downward velocity when the horizontal velocity is in the peak phase but an upward velocity when the horizontal velocity is in the valley phase. In this study, the gusty streamwise and wall-normal velocity components at the downstream measuring points were also obtained by low-pass filtering of the raw streamwise and wall-normal velocity data with a cut-off length of *UT*, where *T* is the period of the gusty wind inflow equal to *T*_*b*_ (60 s). As an example, the gusty streamwise and wall-normal velocity components at *z*/*δ* = 0.50 for both steady inflow and gusty wind inflow are shown in Fig. 8. There are considerable wall-normal airflows induced by gusty wind inflow, and the gusty wall-normal velocity and streamwise velocity are negatively related, consistent with the conclusion of Zeng *et al*.^31^.

**Figure 9:**
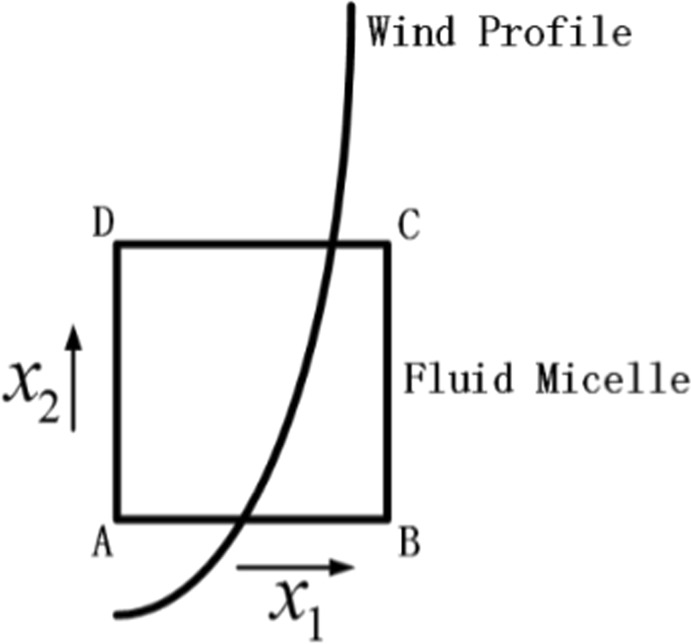
Sketch map of fluid micelle ABCD.

In theory, because the inlet wind profile presents the logarithmic distribution, the wind speed variation at the higher position would be larger than that at the lower position when inflow horizontal wind speed changes, which would cause the vertical distribution of the downstream horizontal wind field to no longer meet logarithmic law. To regain the logarithmic distribution, vertical airflow exchange is essential. We introduced the concept of fluid micelles to explain the mechanism of the production of macroscopic vertical flows from the viewpoint of fluid mechanics. For the fluid micelle ABCD shown in Fig. 9, we assume that the velocity of points A and B are *u* along the horizontal direction marked as *x*_1_. The velocity at points C and D can be described as $u+\frac{\partial u}{\partial {x}_{2}}d{x}_{2}$, where *x*_2_ represents the vertical direction. Under conditions of gusty horizontal wind inflow, the velocity at points A and B are changed to $u+\frac{\partial u}{\partial t}dt$ after *dt*, while the velocity at points C and D become $u+\frac{\partial u}{\partial {x}_{2}}d{x}_{2}+\frac{\partial u}{\partial t}\frac{u+\frac{\partial u}{\partial {x}_{2}}d{x}_{2}}{u}dt$. Therefore, during the period of *dt*, the displacement difference between CD and AB (Δ*s*) can be described by the following equation:

4 $${\rm{\Delta }}s=\frac{\partial u}{\partial {x}_{2}}d{x}_{2}dt+\frac{1}{2}\frac{\partial u}{\partial t}\frac{\frac{\partial u}{\partial {x}_{2}}d{x}_{2}}{u}dtdt$$

By simplifying Eq. (4), we acquired Eq. (5):

5 $${\rm{\Delta }}s={\rm{\Delta }}udt+\frac{1}{2}du\frac{{\rm{\Delta }}u}{u}dt$$

where Δ*u* represents the spatial variation of horizontal velocity, and *du* represents the time variation of horizontal velocity. Because of the displacement difference mentioned above, there is a vertical transport of momentum in the flow field. In other words, there are macroscopic vertical airflows induced by gusty horizontal wind inflows. Furthermore, it is conceivable that considerable vertical airflows would be induced by horizontal gusty wind in atmospheric boundary layer, which would play a key role in the vertical transport of matter.

### The influence of the average value

**Figure 10:**
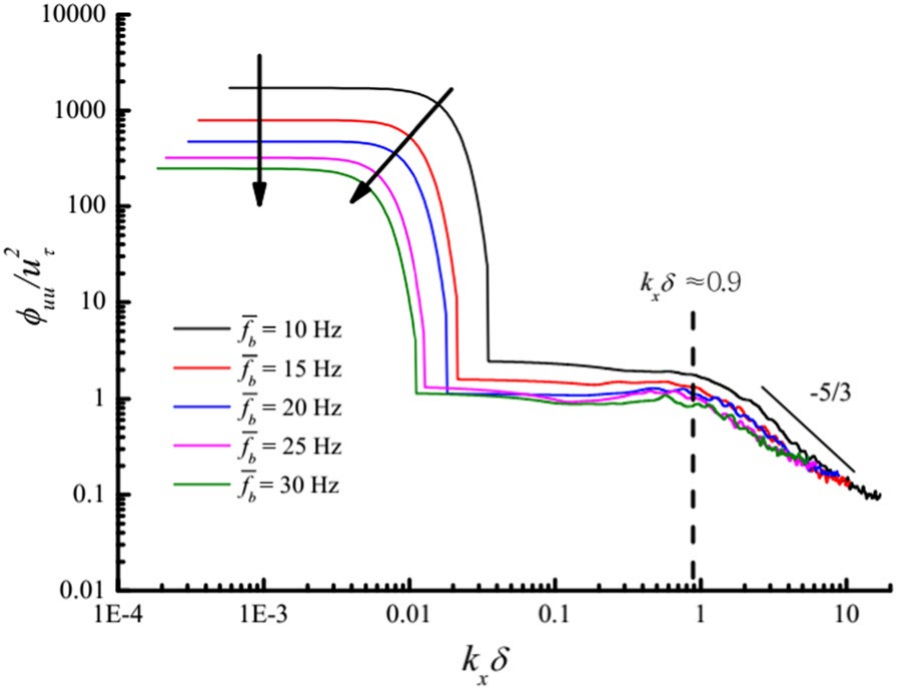
Power spectra of streamwise velocity fluctuations at a height of 0.15 m for different average values.

To explore the influence of average value of the gusty wind inflow, the frequencies of both blowers were synchronously changed according to a sine law with five different average values $\overline{{f}_{b}}$ (10 Hz, 15 Hz, 20 Hz, 25 Hz, and 30 Hz), constant amplitude *A*_*b*_ = 5 Hz, and period *T*_*b*_ = 60 s. The streamwise and wall-normal velocity components at the height of 0.15 m were measured by streamline CTA. After the de-trending process described in Sec. 4.1, the power spectra of five sets of gusty wind inflow were also estimated by Welch average periodogram method as illustrated in Fig. 10. VLSMs and GSMs were detected under all five gusty wind inflows. The average value of the gusty wind inflow has no effect on streamwise wavelength of VLSMs with a constant value of 7*δ* but has a tiny influence on the turbulent energy of the VLSMs. By contrast, the streamwise wavelength of the GSMs *λ*_*GSM*_ tends to increase as the average value because the streamwise wavelength of sinusoidal inflows *λ*_*I*_ = *UT* increases with $\overline{{f}_{b}}$. The increase in $\overline{{f}_{b}}$ would also lead to a gradual decrease in the turbulent energy of the GSMs *E*_*GSM*_.

**Figure 11:**
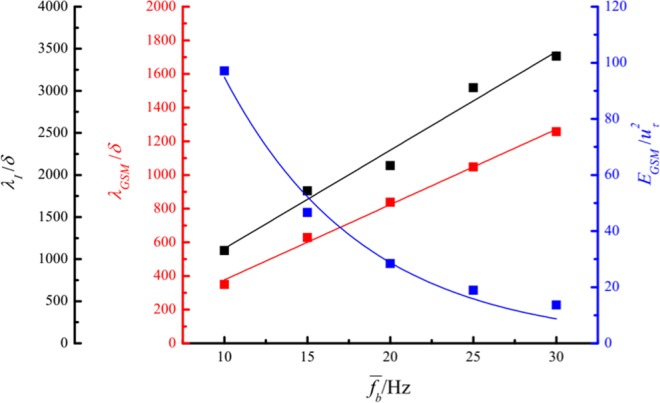
*λ*_*I*_, *λ*_*GSM*_ and *E*_*GSM*_ versus $\overline{{f}_{b}}$ at a height of 0.15 m: the black squares represent the normalized streamwise wavelength of gusty wind inflows; the red squares represent the normalized streamwise wavelength of GSMs; the blue squares represent the normalized turbulent energy of GSMs.

The specific change rules of *λ*_*I*_, *λ*_*GSM*_ and *E*_*GSM*_ versus $\overline{{f}_{b}}$ are described in Fig. 11. The normalized *λ*_*GSM*_ increases linearly with $\overline{{f}_{b}}$, identical to the relationship between *λ*_*I*_ and $\overline{{f}_{b}}$ but with a smaller slope. It is expressed by

6 $$\frac{{\lambda }_{GSM}}{\delta }=-\,70.21+44.72\,{f}_{b}$$

In addition, the normalized *E*_*GSM*_ decreases as a function of $\overline{{f}_{b}}$ according to an exponential law expressed as

7 $$\frac{{E}_{GSM}}{{u}_{\tau }^{2}}=312.41\times {0.89}^{{f}_{b}}$$

According to Eq. (3), the variation in the normalized streamwise wavelength of GSMs and normalized turbulent energy of GSMs versus the local average value of gusty wind inflow *U*_*ht*15_ can be described as

8 $$\frac{{\lambda }_{GSM}}{\delta }=-\,96.68+91.27{U}_{ht15}$$

9 $$\frac{{E}_{GSM}}{{u}_{\tau }^{2}}=334.49\times {0.89}^{\frac{{U}_{ht15}}{0.49}}$$

**Figure 12:**
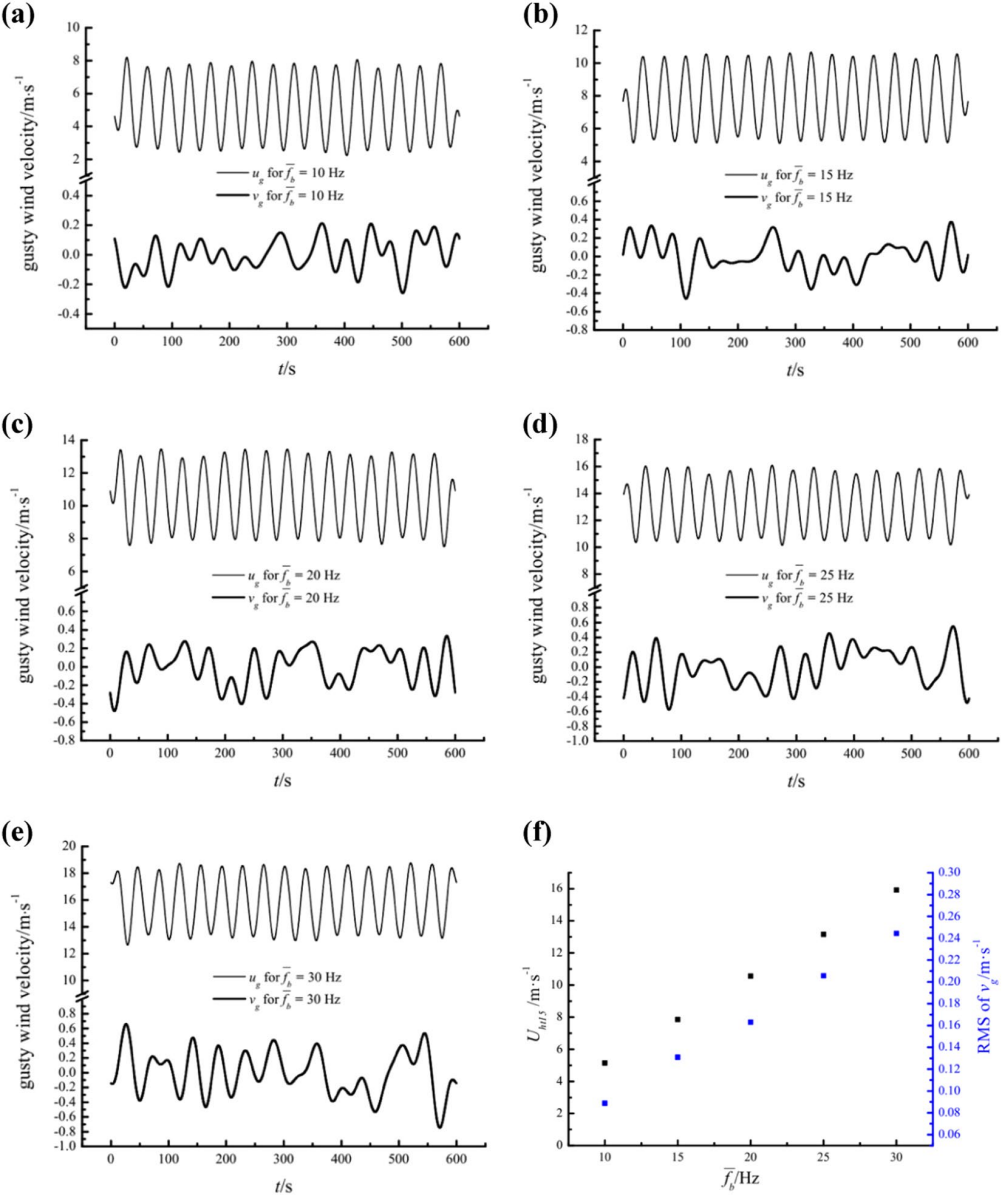
The gusty streamwise and wall-normal velocity components at a height of 0.15 m for different $\overline{{f}_{b}}$ values and variation of the mean streamwise velocity and RMS of the gusty wall-normal velocity versus $\overline{{f}_{b}}$.

Using the method described in Sec. 4.1, the gusty streamwise and wall-normal velocity components for different $\overline{{f}_{b}}$ values at a height of 0.15 m are acquired as illustrated in Fig. 12(a–e). Considerable wall-normal airflows are detected for all $\overline{{f}_{b}}$, and the wall-normal gusty disturbances are negatively correlated with the change in streamwise gusty wind velocity. Of course, this negative correlation is not perfect due to the interference of turbulence. In addition, the RMS values of the gusty wall-normal velocity are sensitive to the local mean streamwise velocity *U*_*ht*15_. The detailed relationship between RMS values of *v*_*g*_ and *U*_*ht*15_ is a perfect linear relationship as shown in Fig. 12(f).

### The influence of amplitude

**Figure 13:**
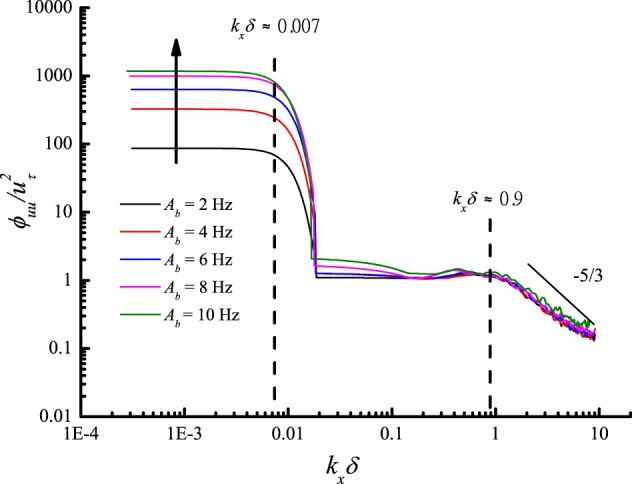
Power spectra of streamwise velocity fluctuations at a height of 0.15 m for different amplitudes.

**Figure 14:**
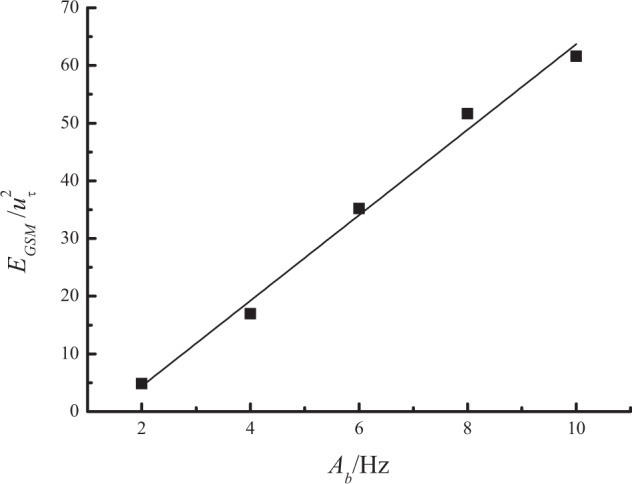
Variation in *E*_*GSM*_ versus *A*_*b*_ at a height of 0.15 m.

By adjusting the running frequencies of both blowers synchronously according to a sine law with five different amplitudes *A*_*b*_ (2 Hz, 4 Hz, 6 Hz, 8 Hz, 10 Hz) and constant average value $\overline{{f}_{b}}$ = 20 Hz, period *T*_*b*_ = 60 s, the influence of amplitude was also studied. The height of the measuring point was remained at 0.15 m. The power spectra of de-trended streamwise velocity fluctuations for five sets of gusty wind inflow are shown in Fig. 13. The VLSMs and GSMs are detected for all gusty wind inflows. The streamwise wavelength and turbulent energy of VLSMs are also independent of *A*_*b*_. Because *λ*_*I*_ remains unchanged for all five gusty wind inflows, *λ*_*GSM*_ is constant approximately 890*δ* for different *A*_*b*_ values. In addition, an increase in *A*_*b*_ will lead to a linear increase in normalized *E*_*GSM*_, as specifically described in Fig. 14. This relationship is expressed by

10 $$\frac{{E}_{GSM}}{{u}_{\tau }^{2}}=-\,10.42+7.41\,{A}_{b}$$

Due to the proportional relation between the rotational speed and running frequency of the blowers, the amplitude of the blowers’ running frequency can also be converted into the amplitude of the local streamwise wind speed by referring to Eq. (3). Then, the relationship between the normalized *E*_*GSM*_ and the amplitude of the local streamwise wind speed, *A*_*ht*15_, is obtained as

11 $$\frac{{E}_{GSM}}{{u}_{\tau }^{2}}=-\,14.81+15.12\,{A}_{ht15}$$

**Figure 15:**
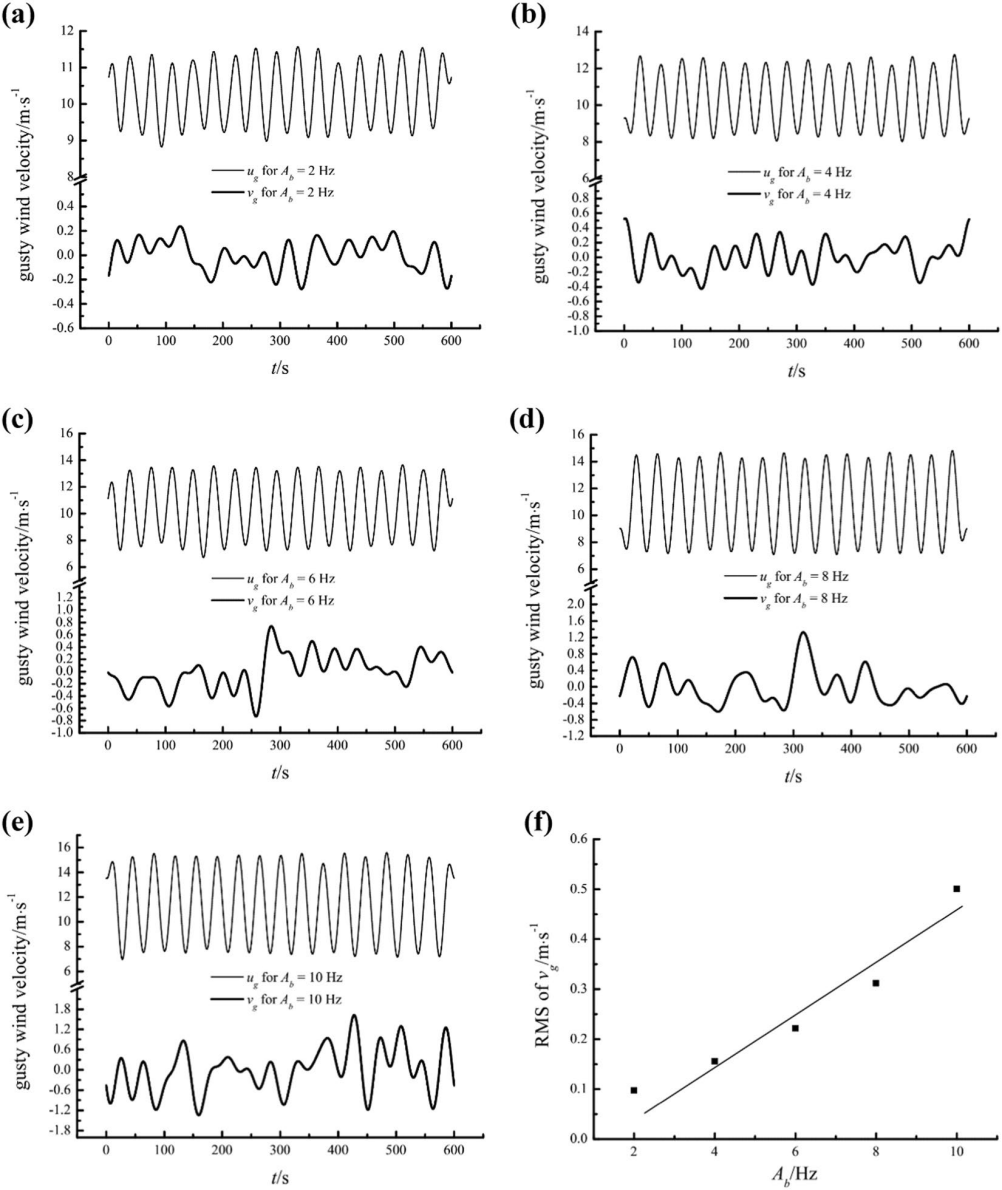
The gusty streamwise and wall-normal velocity components at a height of 0.15 m for different *A*_*b*_ values and the variation in the RMS of the gusty wall-normal velocity versus *A*_*b*_.

Figure 15(a–e) shows the gusty streamwise and wall-normal velocity components for different *A*_*b*_ values at a height of 0.15 m. Gusty wind inflow induces considerable wall-normal airflows that are negatively related to the gusty streamwise velocity for all *A*_*b*_. Furthermore, the RMS values of *v*_*g*_ linearly increase with *A*_*b*_, as illustrated in Fig. 15(f).

### The influence of period

**Figure 16:**
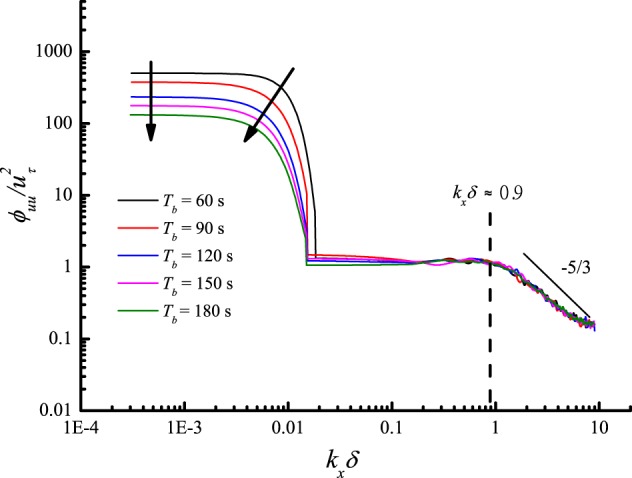
Power spectra of streamwise velocity fluctuations at a height of 0.15 m for different amplitudes.

**Figure 17:**
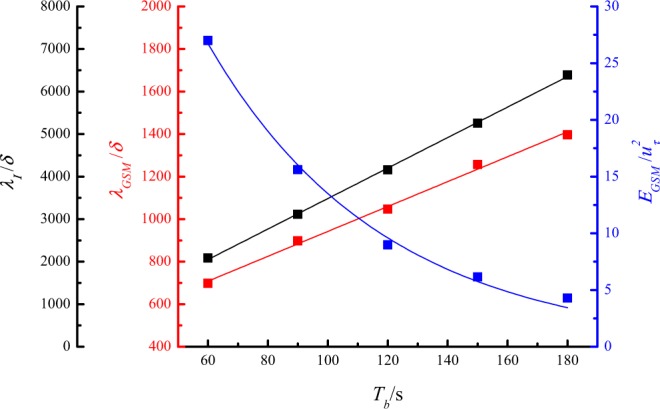
*λ*_*I*_, *λ*_*GSM*_ and *E*_*GSM*_ versus *T*_*b*_ at a height of 0.15 m: the black squares represent the normalized streamwise wavelength of the gusty wind inflows; the red squares represent the normalized streamwise wavelength of the GSMs; the blue squares represent the normalized turbulent energy of the GSMs.

To study the influence of the period of the sinusoidal gusty wind inflow, the running frequencies of both blowers were adjusted synchronously according to a sine law with a constant average value $\overline{{f}_{b}}$ = 20 Hz, amplitude *A*_*b*_ = 5 Hz, and five different periods *T*_*b*_ (60 s, 90 s, 120 s, 150 s, and 180 s). The streamwise and wall-normal velocity components were also measured at the height of 0.15 m. The power spectra of the de-trended streamwise velocity measured under gusty wind inflows with different *T*_*b*_ were estimated and are shown in Fig. 16. As expected, the VLSMs and GSMs were once again detected under gusty wind inflows with different *T*_*b*_. The streamwise wavelength and turbulent energy of the VLSMs were independent of *T*_*b*_ as well. The specific variations of *λ*_*I*_, *λ*_*GSM*_ and *E*_*GSM*_ versus *T*_*b*_ are described in Fig. 17. With the increasing of *T*_*b*_, *λ*_*I*_ linearly increases, resulting in a further linear increase in *λ*_*GSM*_ but with a smaller slope. The similar variations of *λ*_*I*_, *λ*_*GSM*_ versus *T*_*b*_ is a strong support for the conjecture of the formation mechanism of the GSMs. The relationship between normalized *λ*_*GSM*_ and *T*_*b*_ is expressed as

12 $$\frac{\,{\lambda }_{GSM}}{\delta }=357.20+5.85{T}_{b}$$

In addition, the normalized *E*_*GSM*_ decline versus *T*_*b*_ according to an exponential law described by

13 $$\frac{{E}_{GSM}}{{u}_{\tau }^{2}}=74.38\times {0.98}^{{T}_{b}}$$

As a matter of course, the period of gusty wind inflow *T* is equal to *T*_*b*_. The variation in the normalized streamwise wavelength of GSMs and the normalized turbulent energy of the GSMs versus *T* can be expressed as

14 $$\frac{{\lambda }_{GSM}}{\delta }=357.20+5.85T$$

15 $$\frac{{E}_{GSM}}{{u}_{\tau }^{2}}=74.38\times {0.98}^{T}$$

**Figure 18:**
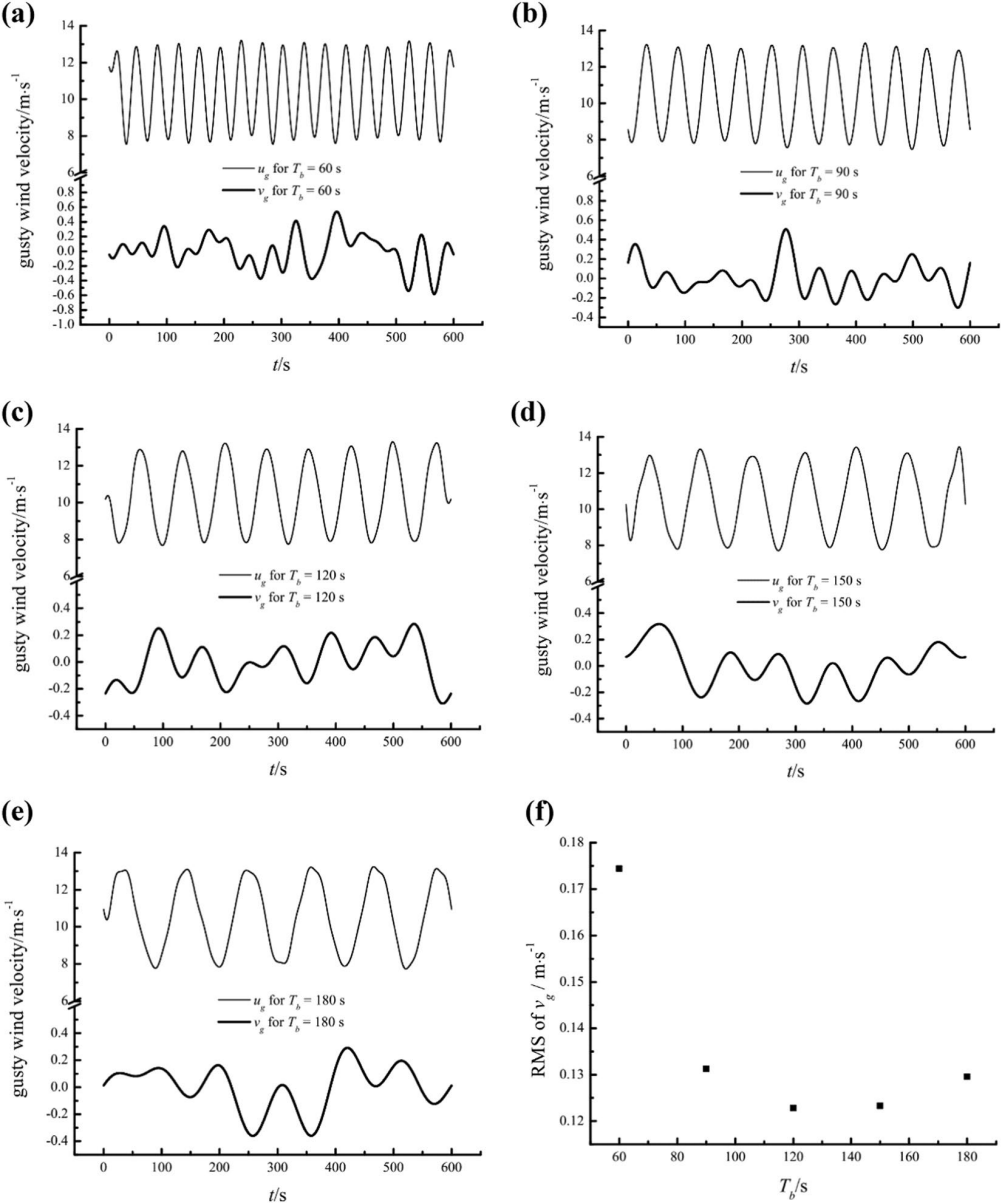
The gusty streamwise and wall-normal velocity components at a height of 0.15 m for different *T*_*b*_ values and variation in the RMS of the gusty wall-normal velocity versus *T*_*b*_.

Figure 18(a–e) show the gusty streamwise and wall-normal velocity components for different *T*_*b*_ values at a height of 0.15 m. Considerable wall-normal airflows are induced by gusty wind inflow and are negatively related to the gusty streamwise velocity for all *T*_*b*_ to some extent. Nevertheless, the relationship between the RMS values of *v*_*g*_ and *T*_*b*_ seems to be ruleless, as illustrated in Fig. 18(f).

In summary, the normalized streamwise wavelength of GSMs in a turbulent boundary layer is linearly related to the average value and period of the sinusoidal gusty wind inflow. This relationship is specifically expressed as

16 $${(\frac{{\lambda }_{GSM}}{\delta })}_{h}={\rm{A}}+{\rm{B}}\times {(U)}_{h}+{\rm{C}}\times T$$

where the subscript *h* represents a certain height in the turbulent boundary layer; *U* is the average value of the sinusoidal gusty wind inflow; *T* is the period of the sinusoidal gusty wind inflow; and A, B, and C are coefficients that depend on the wall-normal height of the measuring point.

Taking logarithms on both sides of Eqs (9), (11) and (15), the logarithmic normalized turbulent energy of GSMs in the turbulent boundary layer is linearly related to the average value, logarithmic amplitude and period of the sinusoidal gusty wind inflow. This relationship is specifically expressed as

17 $${[\mathrm{ln}(\frac{{E}_{GSM}}{{u}_{\tau }^{2}})]}_{h}={\rm{D}}+{\rm{E}}\times {(U)}_{h}+{\rm{F}}\times \,\mathrm{ln}(A)+{\rm{G}}\times T$$

where *A* is the amplitude of the sinusoidal gusty wind inflow and D, E, F, and G are coefficients that depend on the wall-normal height of the measuring point.

Furthermore, any set of wind speed data can be decomposed into multiple sets of sinusoidal wind speed data. While, further research is required to determine whether the features of GSMs under a random gusty wind inflow can be regarded as the superposition of the results of sinusoidal wind inflow.

## Conclusions

Experimental research on turbulence structures and features of a wind field in a straight-through wind tunnel were carried out under steady inflow and gusty wind inflows for the first time in this study. The streamwise and wall-normal velocity components in the laboratory-scale turbulent boundary layer were measured by a streamline CTA equipped with an X-array dual-sensor wire probe Dantec 55P61. Power spectrum analysis confirmed the occurrences of the VLSMs under both steady inflow and gusty wind inflows. The streamwise wavelengths of the VLSMs were fixed (~7*δ*) irrespective of the inflow conditions. The turbulent energy of the VLSMs is dependent on the wall-normal distance between the measuring points and the ground and on the local mean streamwise velocity to some extent. These findings fill in the gap to describe the features of VLSMs under gusty wind conditions.

In addition to VLSMs, turbulence structures with much larger streamwise wavelengths and turbulent energies were detected under gusty wind inflows. Because their streamwise wavelengths are of a similar order of magnitude as the streamwise wavelength of gusty wind inflows, these turbulence structures are named GSMs. The discovery of GSMs under gusty wind conditions is a significant contribution to the study on the turbulence structures. The streamwise wavelength of GSMs increases linearly with the average value and period of the sinusoidal gusty wind inflows, identical to the variation in the inflow streamwise wavelength as a function of both characteristic parameters. These similarities provide conclusive evidence that GSMs originate from regular gusty wind inflows.

GSMs might originate from an ordered external driving force that forces hairpin packets or/and very large scale turbulence structures to align coherently in groups with a length scale related to the gust inflow condition. This is a plausible conjecture of the formation of GSMs and will need further research to confirm by visual approach, such as Particle Image Velocimetry (PIV). The turbulent energy of GSMs is sensitive to the wall-normal height and all characteristic parameters of gusty wind inflows, including the average value, amplitude and period. While, the physical mechanism of the variation of *E*_*GSMs*_ versus the average value, amplitude and period of gusty wind will still need to be further research.

Furthermore, there are considerable vertical airflows induced by gusty wind inflow. The velocity of these airflows is typically downward when the horizontal velocity is in the peak phase but upward when the horizontal velocity is in the valley phase. The RMS values of the gusty wall-normal velocity tend to increase linearly with the average value and amplitude of the gusty wind inflows. In the atmospheric boundary layer, the vertical airflows would play a key role in the vertical transportation of mass and energy.
